# 7-(Benzyl­sulfan­yl)-5-(2-methoxy­phen­yl)-1,3-dimethyl-5,6-dihydro­pyrimido[4,5-*d*]pyrimidine-2,4(1*H*,3*H*)-dione

**DOI:** 10.1107/S160053680802401X

**Published:** 2008-08-06

**Authors:** Ayoob Bazgir, Fereshteh Faraji

**Affiliations:** a Department of Chemistry, Islamic Azad University, Dorood Branch, Dorood 688173551, Iran; b Department of Chemistry, Faculty of Science, Islamic Azad University, Karaj Branch, Karaj, Iran

## Abstract

In the mol­ecule of the title compound, C_22_H_22_N_4_O_3_S, the benzene and phenyl rings are oriented at a dihedral angle of 88.72 (4)°. The other two rings have flattened-boat conformations. In the crystal structure, inter­molecular N—H⋯O hydrogen bonds link the mol­ecules.

## Related literature

For general background, see: Sharma *et al.* (2004 ▶); Quiroga *et al.* (2002 ▶); Devi *et al.* (2003 ▶). For bond-length data, see: Allen *et al.* (1987 ▶). For ring conformation puckering parameters, see: Cremer & Pople (1975 ▶).
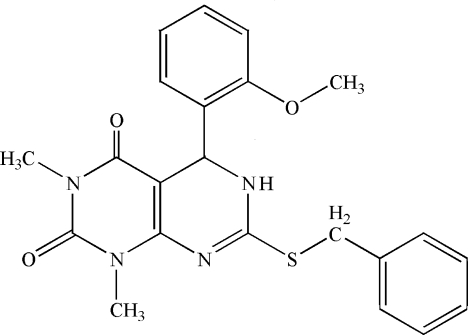


## Experimental

### 

#### Crystal data

C_22_H_22_N_4_O_3_S
*M* *_r_* = 422.51
Monoclinic, 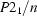

*a* = 10.9216 (9) Å
*b* = 8.8528 (5) Å
*c* = 20.7263 (15) Å
β = 90.638 (6)°
*V* = 2003.8 (2) Å^3^
*Z* = 4
Mo *K*α radiation
μ = 0.20 mm^−1^
*T* = 294 (2) K
0.4 × 0.3 × 0.05 mm

#### Data collection

Bruker SMART CCD area-detector diffractometer
Absorption correction: multi-scan (*SADABS*; Sheldrick, 1998 ▶) *T*_min_ = 0.928, *T*_max_ = 0.985
23191 measured reflections
5394 independent reflections
4510 reflections with *I* > 2σ(*I*)
*R*_int_ = 0.063

#### Refinement

*R*[*F* ^2^ > 2σ(*F* ^2^)] = 0.055
*wR*(*F* ^2^) = 0.135
*S* = 1.15
5394 reflections
278 parameters
H atoms treated by a mixture of independent and constrained refinement
Δρ_max_ = 0.32 e Å^−3^
Δρ_min_ = −0.25 e Å^−3^

### 

Data collection: *SMART* (Bruker, 1998 ▶); cell refinement: *SAINT* (Bruker, 1998 ▶); data reduction: *SAINT*; program(s) used to solve structure: *SHELXTL* (Sheldrick, 2008 ▶); program(s) used to refine structure: *SHELXTL*; molecular graphics: *ORTEP-3 for Windows* (Farrugia, 1997 ▶); software used to prepare material for publication: *WinGX* (Farrugia, 1999 ▶).

## Supplementary Material

Crystal structure: contains datablocks global, I. DOI: 10.1107/S160053680802401X/hk2501sup1.cif

Structure factors: contains datablocks I. DOI: 10.1107/S160053680802401X/hk2501Isup2.hkl

Additional supplementary materials:  crystallographic information; 3D view; checkCIF report

## Figures and Tables

**Table 1:** Hydrogen-bond geometry (Å, °)

*D*—H⋯*A*	*D*—H	H⋯*A*	*D*⋯*A*	*D*—H⋯*A*
N4—H4*B*⋯O1^i^	0.85 (3)	2.02 (3)	2.836 (2)	161 (2)

Symmetry code: (i) 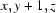.

**Fig. 1.**
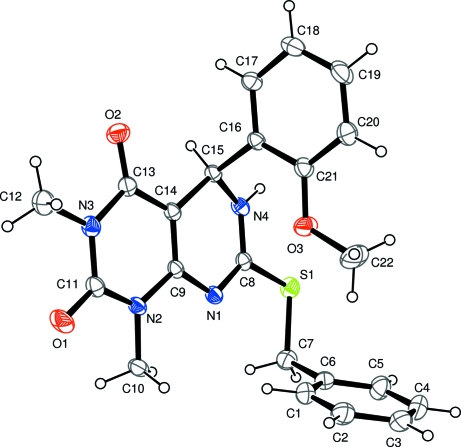
The molecular structure of the title molecule, with the atom-numbering scheme. Displacement ellipsoids are drawn at the 30% probability level.

**Fig. 2.**
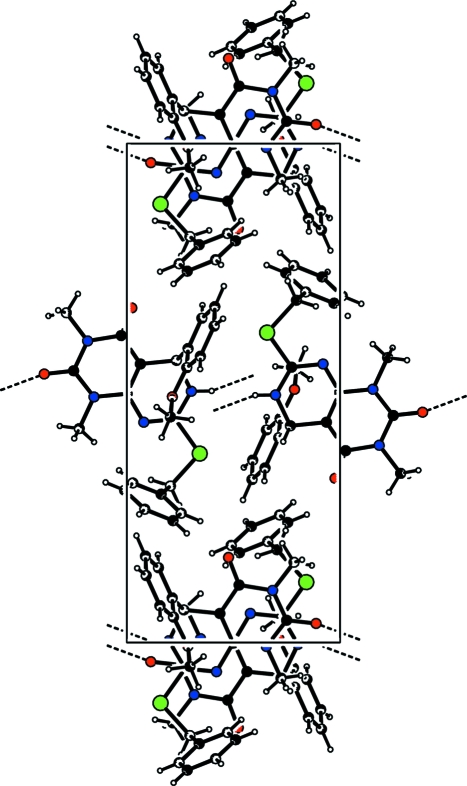
A packing diagram of the title compound. Hydrogen bonds are shown as dashed lines.

## supplementary crystallographic information

### Comment

The importance of fused pyrimidines, common source for the development of new potential therapeutic agents (Sharma *et al.*,  2004), is well known. Among them, the pyrimido[2,3-d]pyrimidines are an important class of annulated uracils with biological significance because of their connection with purine pteridine system (Quiroga *et al.*,  2002). Numerous reports delineate the antitumor, antivial, antioxidant, antifungal and hepatoprotective activities of these compounds (Devi *et al.*,  2003). Therefore, for the preparation of these complex molecules large efforts have been directed towards the synthetic manipulation of uracils. We report herein the synthesis and crystal structure of the title compound.

In the molecule of the title compound (Fig. 1), the bond lengths (Allen *et al.*,  1987) and angles are within normal ranges. Rings A (C1-C6) and D (C16-C21) are, of course, planar and they are oriented at a dihedral angle of A/D = 88.72 (4)°. Rings  B (N2/N3/C9/C11/C13/C14) and C (N1/N4/C8/C9/C14/C15) have flattened-boat [φ = 105.63 (2)°, θ = 100.53 (3)° (for ring B) and φ = 29.58 (3)°, θ = 58.23 (3)° (for ring C)] conformations, having total puckering amplitudes, Q_T_, of 0.120 (3) and 0.364 (3) Å, respectively (Cremer & Pople, 1975).

In the crystal structure, intermolecular N-H···O hydrogen bonds (Table 1) link the molecules (Fig. 2), in which they may be effective in the stabilization of the structure.

### Experimental

For the preparation of the title compound, 6-amino-1,3-dimethyluracil (0.15 g, 1 mmol), 2-methylbenzaldehyde (0.12 g, 1 mmol), 2-benzylthiourea hydrochloride (0.30 g, 1.5 mmol) and p-toluenesulfonic acid (0.1 g) were mixed. The reaction mixture was placed in a screw capped vial and irradiated for 5 min with a power of 700 W microwave irradiation. After cooling, the reaction mixture was washed with water, and then recrystallized from ethyl acetate to afford the title compound (yield; 0.25 g. 65%, m.p. 519-521 K).

### Refinement

H4B atom (for NH) was located in difference syntheses and refined isotropically [N-H = 0.85 (3) Å and U_iso_(H) = 0.049 (6) Å^2^]. The remaining H atoms were positioned geometrically, with C-H = 0.93, 0.98, 0.97 and 0.96 Å for aromatic, methine, methylene and methyl H, respectively, and constrained to ride on their parent atoms with U_iso_(H) = xU_eq_(C), where x = 1.5 for methyl H and x = 1.2 for all other H atoms.

### Crystal data

**Table:**

C_22_H_22_N_4_O_3_S	*F*_000_ = 888
*M**_r_* = 422.51	*D*_x_ = 1.401 Mg m^−^^3^
Monoclinic, *P*2_1_/*n*	Mo *K*α radiation λ = 0.71073 Å
Hall symbol: -P 2yn	Cell parameters from 2175 reflections
*a* = 10.9216 (9) Å	θ = 2.1–29.3º
*b* = 8.8528 (5) Å	µ = 0.20 mm^−^^1^
*c* = 20.7263 (15) Å	*T* = 294 (2) K
β = 90.638 (6)º	Plate, colorless
*V* = 2003.8 (2) Å^3^	0.4 × 0.3 × 0.05 mm
*Z* = 4	

### Data collection

**Table:**

Bruker SMART CCD area-detector diffractometer	*R*_int_ = 0.063
φ and ω scans	θ_max_ = 29.3º
Absorption correction: multi-scan(SADABS; Sheldrick, 1998)	θ_min_ = 2.1º
*T*_min_ = 0.928, *T*_max_ = 0.985	*h* = −14→15
23191 measured reflections	*k* = −11→12
5394 independent reflections	*l* = −28→28
4510 reflections with *I* > 2σ(*I*)	

### Refinement

**Table:**

Refinement on *F*^2^	H atoms treated by a mixture of independent and constrained refinement
Least-squares matrix: full	*w* = 1/[σ^2^(*F*_o_^2^) + (0.0538*P*)^2^ + 0.6815*P*] where *P* = (*F*_o_^2^ + 2*F*_c_^2^)/3
*R*[*F*^2^ > 2σ(*F*^2^)] = 0.055	(Δ/σ)_max_ = 0.038
*wR*(*F*^2^) = 0.135	Δρ_max_ = 0.32 e Å^−^^3^
*S* = 1.15	Δρ_min_ = −0.25 e Å^−^^3^
5394 reflections	Extinction correction: none
278 parameters	

### Special details

**Table:**

Geometry. All e.s.d.'s (except the e.s.d. in the dihedral angle between two l.s. planes) are estimated using the full covariance matrix. The cell e.s.d.'s are taken into account individually in the estimation of e.s.d.'s in distances, angles and torsion angles; correlations between e.s.d.'s in cell parameters are only used when they are defined by crystal symmetry. An approximate (isotropic) treatment of cell e.s.d.'s is used for estimating e.s.d.'s involving l.s. planes.

### Fractional atomic coordinates and isotropic or equivalent isotropic displacement parameters (Å^2^)

**Table:**

	*x*	*y*	*z*	*U*_iso_*/*U*_eq_	
S1	0.34470 (5)	0.84201 (5)	0.12120 (2)	0.04173 (13)	
O1	0.34328 (15)	0.11232 (15)	−0.03830 (7)	0.0508 (4)	
O2	0.32309 (14)	0.52289 (16)	−0.17071 (6)	0.0469 (3)	
O3	0.08733 (11)	0.71339 (19)	0.00741 (7)	0.0502 (4)	
N1	0.33713 (13)	0.57943 (16)	0.05837 (7)	0.0322 (3)	
N2	0.34856 (13)	0.34559 (15)	0.00770 (7)	0.0320 (3)	
N3	0.32991 (14)	0.31591 (16)	−0.10452 (7)	0.0349 (3)	
N4	0.33864 (13)	0.80243 (16)	−0.00370 (7)	0.0326 (3)	
H4B	0.341 (2)	0.898 (3)	−0.0043 (11)	0.049 (6)*	
C1	0.15207 (19)	0.5279 (2)	0.18010 (10)	0.0459 (4)	
H1	0.1982	0.4632	0.1547	0.055*	
C2	0.0325 (2)	0.4896 (3)	0.19479 (10)	0.0509 (5)	
H2	−0.0011	0.4002	0.179	0.061*	
C3	−0.0368 (2)	0.5839 (3)	0.23278 (10)	0.0526 (5)	
H3	−0.1169	0.5582	0.2429	0.063*	
C4	0.0136 (2)	0.7165 (3)	0.25561 (11)	0.0592 (6)	
H4	−0.0328	0.7807	0.2811	0.071*	
C5	0.1329 (2)	0.7546 (3)	0.24080 (10)	0.0525 (5)	
H5	0.1659	0.8443	0.2566	0.063*	
C6	0.20406 (17)	0.6607 (2)	0.20257 (8)	0.0388 (4)	
C7	0.33376 (18)	0.7042 (2)	0.18617 (9)	0.0432 (4)	
H7A	0.3782	0.6139	0.1739	0.052*	
H7B	0.3733	0.7453	0.2245	0.052*	
C8	0.33809 (14)	0.72697 (18)	0.05202 (8)	0.0300 (3)	
C9	0.33441 (13)	0.50102 (17)	0.00128 (8)	0.0288 (3)	
C10	0.37101 (17)	0.2765 (2)	0.07119 (9)	0.0382 (4)	
H10A	0.2942	0.2532	0.0909	0.057*	
H10B	0.4159	0.3458	0.0981	0.057*	
H10C	0.4176	0.1854	0.066	0.057*	
C11	0.34047 (15)	0.25006 (18)	−0.04476 (9)	0.0345 (3)	
C12	0.3234 (2)	0.2154 (2)	−0.16086 (10)	0.0483 (5)	
H12A	0.3785	0.1321	−0.1546	0.072*	
H12B	0.3461	0.2704	−0.1988	0.072*	
H12C	0.2414	0.178	−0.166	0.072*	
C13	0.32425 (15)	0.47335 (19)	−0.11530 (8)	0.0331 (3)	
C14	0.31967 (14)	0.56353 (17)	−0.05833 (8)	0.0293 (3)	
C15	0.30427 (14)	0.73269 (17)	−0.06556 (8)	0.0287 (3)	
H15	0.3644	0.7662	−0.0973	0.034*	
C16	0.17931 (14)	0.78714 (18)	−0.08936 (8)	0.0301 (3)	
C17	0.16945 (17)	0.8519 (2)	−0.15010 (9)	0.0378 (4)	
H17	0.2381	0.8546	−0.1762	0.045*	
C18	0.0604 (2)	0.9128 (2)	−0.17325 (10)	0.0469 (5)	
H18	0.0559	0.9551	−0.2143	0.056*	
C19	−0.0411 (2)	0.9096 (3)	−0.13438 (11)	0.0532 (5)	
H19	−0.1143	0.9515	−0.1491	0.064*	
C20	−0.03502 (18)	0.8450 (3)	−0.07381 (10)	0.0493 (5)	
H20	−0.1041	0.8439	−0.048	0.059*	
C21	0.07381 (16)	0.7812 (2)	−0.05109 (9)	0.0371 (4)	
C22	−0.0178 (2)	0.7044 (4)	0.04781 (13)	0.0771 (9)	
H22A	−0.0516	0.8035	0.0535	0.116*	
H22B	0.0057	0.6636	0.089	0.116*	
H22C	−0.0781	0.64	0.028	0.116*	

### Atomic displacement parameters (Å^2^)

**Table:**

	*U*^11^	*U*^22^	*U*^33^	*U*^12^	*U*^13^	*U*^23^
C1	0.0512 (11)	0.0413 (10)	0.0454 (10)	0.0063 (8)	0.0071 (8)	−0.0019 (8)
C2	0.0550 (12)	0.0452 (11)	0.0528 (11)	−0.0047 (9)	0.0061 (9)	0.0045 (9)
C3	0.0478 (11)	0.0587 (13)	0.0515 (11)	0.0019 (10)	0.0107 (9)	0.0136 (10)
C4	0.0607 (14)	0.0600 (14)	0.0573 (13)	0.0121 (11)	0.0216 (11)	−0.0024 (11)
C5	0.0591 (13)	0.0489 (12)	0.0495 (11)	−0.0002 (9)	0.0081 (9)	−0.0100 (9)
C6	0.0425 (9)	0.0429 (10)	0.0311 (8)	0.0043 (7)	−0.0013 (7)	0.0027 (7)
C7	0.0402 (9)	0.0522 (11)	0.0372 (9)	0.0014 (8)	−0.0051 (7)	−0.0013 (8)
C8	0.0233 (7)	0.0286 (7)	0.0383 (8)	0.0005 (5)	0.0010 (6)	−0.0006 (6)
C9	0.0228 (7)	0.0239 (7)	0.0398 (8)	0.0006 (5)	0.0039 (6)	0.0030 (6)
C10	0.0377 (9)	0.0320 (8)	0.0447 (9)	0.0034 (7)	0.0009 (7)	0.0115 (7)
C11	0.0318 (8)	0.0251 (7)	0.0467 (9)	−0.0018 (6)	0.0029 (7)	0.0016 (7)
C12	0.0609 (12)	0.0340 (9)	0.0501 (11)	−0.0013 (9)	−0.0015 (9)	−0.0085 (8)
C13	0.0302 (8)	0.0285 (7)	0.0407 (8)	−0.0014 (6)	0.0046 (6)	0.0017 (7)
C14	0.0258 (7)	0.0242 (7)	0.0381 (8)	−0.0002 (5)	0.0027 (6)	0.0029 (6)
C15	0.0262 (7)	0.0242 (7)	0.0356 (8)	−0.0012 (5)	0.0034 (6)	0.0052 (6)
C16	0.0287 (7)	0.0246 (7)	0.0369 (8)	−0.0004 (6)	0.0005 (6)	0.0034 (6)
C17	0.0413 (9)	0.0327 (8)	0.0395 (9)	−0.0009 (7)	0.0007 (7)	0.0054 (7)
C18	0.0540 (12)	0.0421 (10)	0.0444 (10)	0.0064 (9)	−0.0092 (8)	0.0101 (8)
C19	0.0440 (11)	0.0559 (12)	0.0593 (12)	0.0163 (9)	−0.0118 (9)	0.0057 (10)
C20	0.0320 (9)	0.0632 (13)	0.0525 (11)	0.0102 (9)	0.0007 (8)	0.0039 (10)
C21	0.0303 (8)	0.0393 (9)	0.0415 (9)	0.0023 (7)	0.0007 (6)	0.0042 (7)
C22	0.0416 (12)	0.127 (3)	0.0628 (15)	0.0041 (14)	0.0173 (10)	0.0345 (16)
N1	0.0338 (7)	0.0268 (6)	0.0361 (7)	0.0024 (5)	0.0011 (5)	0.0018 (5)
N2	0.0324 (7)	0.0240 (6)	0.0396 (7)	0.0015 (5)	0.0024 (5)	0.0056 (5)
N3	0.0379 (7)	0.0257 (7)	0.0411 (7)	−0.0017 (5)	0.0034 (6)	−0.0025 (6)
N4	0.0340 (7)	0.0220 (6)	0.0418 (8)	−0.0020 (5)	−0.0016 (6)	0.0017 (6)
O1	0.0688 (10)	0.0210 (6)	0.0624 (9)	−0.0018 (6)	−0.0002 (7)	0.0037 (6)
O2	0.0654 (9)	0.0384 (7)	0.0371 (6)	−0.0033 (6)	0.0075 (6)	0.0023 (6)
O3	0.0279 (6)	0.0759 (10)	0.0468 (7)	0.0022 (6)	0.0057 (5)	0.0226 (7)
S1	0.0468 (3)	0.0340 (2)	0.0444 (2)	−0.00253 (18)	0.00230 (19)	−0.00687 (18)

### Geometric parameters (Å, °)

**Table:**

N4—H4B	0.85 (3)	C11—N2	1.379 (2)
C1—C6	1.383 (3)	C12—N3	1.469 (2)
C1—C2	1.387 (3)	C12—H12A	0.96
C1—H1	0.93	C12—H12B	0.96
C2—C3	1.379 (3)	C12—H12C	0.96
C2—H2	0.93	C13—O2	1.229 (2)
C3—C4	1.378 (4)	C13—N3	1.413 (2)
C3—H3	0.93	C13—C14	1.427 (2)
C4—C5	1.383 (3)	C14—C15	1.514 (2)
C4—H4	0.93	C15—N4	1.468 (2)
C5—C6	1.392 (3)	C15—C16	1.524 (2)
C5—H5	0.93	C15—H15	0.98
C6—C7	1.510 (3)	C16—C17	1.386 (2)
C7—S1	1.822 (2)	C16—C21	1.407 (2)
C7—H7A	0.97	C17—C18	1.388 (3)
C7—H7B	0.97	C17—H17	0.93
C8—N1	1.313 (2)	C18—C19	1.377 (3)
C8—N4	1.334 (2)	C18—H18	0.93
C8—S1	1.7596 (17)	C19—C20	1.381 (3)
C9—C14	1.362 (2)	C19—H19	0.93
C9—N1	1.372 (2)	C20—C21	1.393 (3)
C9—N2	1.3908 (19)	C20—H20	0.93
C10—N2	1.469 (2)	C21—O3	1.359 (2)
C10—H10A	0.96	C22—O3	1.431 (2)
C10—H10B	0.96	C22—H22A	0.96
C10—H10C	0.96	C22—H22B	0.96
C11—O1	1.227 (2)	C22—H22C	0.96
C11—N3	1.373 (2)		
			
C6—C1—C2	121.20 (19)	N3—C13—C14	115.02 (14)
C6—C1—H1	119.4	C9—C14—C13	121.24 (14)
C2—C1—H1	119.4	C9—C14—C15	120.24 (14)
C3—C2—C1	120.1 (2)	C13—C14—C15	118.45 (14)
C3—C2—H2	120	N4—C15—C14	107.61 (13)
C1—C2—H2	120	N4—C15—C16	111.66 (13)
C4—C3—C2	119.4 (2)	C14—C15—C16	116.28 (13)
C4—C3—H3	120.3	N4—C15—H15	106.9
C2—C3—H3	120.3	C14—C15—H15	106.9
C3—C4—C5	120.4 (2)	C16—C15—H15	106.9
C3—C4—H4	119.8	C17—C16—C21	118.14 (15)
C5—C4—H4	119.8	C17—C16—C15	119.01 (14)
C4—C5—C6	120.9 (2)	C21—C16—C15	122.79 (14)
C4—C5—H5	119.5	C16—C17—C18	122.16 (17)
C6—C5—H5	119.5	C16—C17—H17	118.9
C1—C6—C5	117.99 (19)	C18—C17—H17	118.9
C1—C6—C7	121.56 (17)	C19—C18—C17	118.85 (18)
C5—C6—C7	120.45 (18)	C19—C18—H18	120.6
C6—C7—S1	113.99 (13)	C17—C18—H18	120.6
C6—C7—H7A	108.8	C18—C19—C20	120.64 (18)
S1—C7—H7A	108.8	C18—C19—H19	119.7
C6—C7—H7B	108.8	C20—C19—H19	119.7
S1—C7—H7B	108.8	C19—C20—C21	120.48 (19)
H7A—C7—H7B	107.6	C19—C20—H20	119.8
N1—C8—N4	125.81 (15)	C21—C20—H20	119.8
N1—C8—S1	119.63 (13)	O3—C21—C20	124.34 (17)
N4—C8—S1	114.52 (12)	O3—C21—C16	115.97 (15)
C14—C9—N1	125.32 (14)	C20—C21—C16	119.68 (17)
C14—C9—N2	120.04 (15)	O3—C22—H22A	109.5
N1—C9—N2	114.64 (14)	O3—C22—H22B	109.5
N2—C10—H10A	109.5	H22A—C22—H22B	109.5
N2—C10—H10B	109.5	O3—C22—H22C	109.5
H10A—C10—H10B	109.5	H22A—C22—H22C	109.5
N2—C10—H10C	109.5	H22B—C22—H22C	109.5
H10A—C10—H10C	109.5	C8—N1—C9	114.65 (14)
H10B—C10—H10C	109.5	C11—N2—C9	121.68 (14)
O1—C11—N3	121.48 (17)	C11—N2—C10	117.34 (14)
O1—C11—N2	121.47 (17)	C9—N2—C10	120.98 (14)
N3—C11—N2	117.05 (14)	C11—N3—C13	124.39 (14)
N3—C12—H12A	109.5	C11—N3—C12	117.56 (15)
N3—C12—H12B	109.5	C13—N3—C12	118.05 (15)
H12A—C12—H12B	109.5	C8—N4—C15	122.81 (14)
N3—C12—H12C	109.5	C8—N4—H4B	120.9 (16)
H12A—C12—H12C	109.5	C15—N4—H4B	114.5 (16)
H12B—C12—H12C	109.5	C21—O3—C22	117.82 (16)
O2—C13—N3	119.99 (16)	C8—S1—C7	102.25 (9)
O2—C13—C14	124.99 (16)		
			
C6—C1—C2—C3	−0.4 (3)	C17—C16—C21—O3	178.46 (16)
C1—C2—C3—C4	0.3 (3)	C15—C16—C21—O3	−4.6 (3)
C2—C3—C4—C5	−0.2 (4)	C17—C16—C21—C20	−2.5 (3)
C3—C4—C5—C6	0.2 (4)	C15—C16—C21—C20	174.39 (17)
C2—C1—C6—C5	0.4 (3)	N4—C8—N1—C9	−1.1 (2)
C2—C1—C6—C7	−179.27 (18)	S1—C8—N1—C9	−178.71 (11)
C4—C5—C6—C1	−0.2 (3)	C14—C9—N1—C8	−7.4 (2)
C4—C5—C6—C7	179.4 (2)	N2—C9—N1—C8	172.47 (14)
C1—C6—C7—S1	100.2 (2)	O1—C11—N2—C9	−174.13 (16)
C5—C6—C7—S1	−79.5 (2)	N3—C11—N2—C9	6.3 (2)
N1—C9—C14—C13	176.78 (15)	O1—C11—N2—C10	5.0 (2)
N2—C9—C14—C13	−3.1 (2)	N3—C11—N2—C10	−174.62 (14)
N1—C9—C14—C15	−0.1 (2)	C14—C9—N2—C11	−4.1 (2)
N2—C9—C14—C15	−179.98 (13)	N1—C9—N2—C11	175.99 (14)
O2—C13—C14—C9	−172.94 (17)	C14—C9—N2—C10	176.78 (15)
N3—C13—C14—C9	7.4 (2)	N1—C9—N2—C10	−3.1 (2)
O2—C13—C14—C15	4.0 (2)	O1—C11—N3—C13	178.99 (17)
N3—C13—C14—C15	−175.67 (14)	N2—C11—N3—C13	−1.4 (2)
C9—C14—C15—N4	13.62 (19)	O1—C11—N3—C12	−0.7 (3)
C13—C14—C15—N4	−163.38 (13)	N2—C11—N3—C12	178.94 (16)
C9—C14—C15—C16	−112.44 (17)	O2—C13—N3—C11	175.12 (16)
C13—C14—C15—C16	70.56 (19)	C14—C13—N3—C11	−5.2 (2)
N4—C15—C16—C17	124.18 (16)	O2—C13—N3—C12	−5.2 (2)
C14—C15—C16—C17	−111.82 (17)	C14—C13—N3—C12	174.47 (15)
N4—C15—C16—C21	−52.7 (2)	N1—C8—N4—C15	17.5 (2)
C14—C15—C16—C21	71.3 (2)	S1—C8—N4—C15	−164.81 (11)
C21—C16—C17—C18	1.3 (3)	C14—C15—N4—C8	−21.9 (2)
C15—C16—C17—C18	−175.73 (17)	C16—C15—N4—C8	106.81 (17)
C16—C17—C18—C19	0.4 (3)	C20—C21—O3—C22	1.3 (3)
C17—C18—C19—C20	−1.0 (3)	C16—C21—O3—C22	−179.8 (2)
C18—C19—C20—C21	−0.3 (4)	N1—C8—S1—C7	−5.17 (15)
C19—C20—C21—O3	−179.0 (2)	N4—C8—S1—C7	176.99 (12)
C19—C20—C21—C16	2.1 (3)	C6—C7—S1—C8	−84.81 (15)

### Hydrogen-bond geometry (Å, °)

**Table:**

*D*—H···*A*	*D*—H	H···*A*	*D*···*A*	*D*—H···*A*
N4—H4B···O1^i^	0.85 (3)	2.02 (3)	2.836 (2)	161 (2)

Symmetry codes: (i) *x*, *y*+1, *z*.
